# Partial hypopituitarism with ACTH deficiency as the main manifestation as a complication of hemorrhagic fever with renal syndrome

**DOI:** 10.1186/s12902-024-01587-4

**Published:** 2024-05-07

**Authors:** Shaomin Shi, Aoni Zhang, Jingjing Zhang, Shaoyong Xu

**Affiliations:** 1 Department of Endocrinology, Xiangyang Central Hospital, Affiliated Hospital of Hubei University of Arts and Science, 139 JingzhouStreet, Xiangyang, Hubei 441000 China; 2 Center for Clinical Evidence-Based and Translational Medicine, Xiangyang Central Hospital, Affiliated Hospital of Hubei University of Arts and Science, Xiangyang, Hubei China

**Keywords:** Hypopituitarism, Hemorrhagic fever with renal syndrome (HFRS), ACTH deficiency, Fatigue

## Abstract

Hypopituitarism is a relatively rare complication of hemorrhagic fever with renal syndrome. However, almost all available reported cases were total anterior pituitary hypofunction, isolated growth-hormone deficiency, or isolated gonadotropin deficiency. Here, we firstly describe a patient with partial hypopituitarism with ACTH deficiency as the main manifestation as a complication of hemorrhagic fever with renal syndrome.

## Introduction

Hemorrhagic fever with renal syndrome (HFRS) is an acute natural epidemic disease caused by Hantavirus, and rodents are the main source of infection via aerosols from urine, saliva, and feces [1, 2]. It occurs either sporadically or in epidemics (the last one in 1995). Its main pathological changes include extensive injury to systemic small blood vessels and capillaries, with fever, shock, congestion, bleeding and renal failure as the main clinical characteristics. The five characteristic phases are febrile, hypotensive, oliguric, diuretic, and convalescent [3]. HFRS were first reported to have spread during the second World War [4], and military personnel are at high risk [5, 6]. Its clinical manifestations vary in severity. Most patients can be cured, but some patients have complications [7]. Hypopituitarism is a relatively rare complication. These reports suggest that patients with HFRS may develop hypopituitarism via concomitant pituitary hemorrhage and subsequent atrophy. However, almost all available reported cases were total anterior pituitary hypofunction, isolated growth-hormone deficiency, or isolated gonadotropin deficiency. Here, we describe a patient with partial hypopituitarism with ACTH deficiency as the main manifestation as a complication of HFRS.

## Case report

A 55-year-old man presented to our department with fatigue for 2 years, repeated nausea, and poor appetite for more than 20 days. Two years before his visit to our hospital (November 2021), he had contact with a dead mouse and was diagnosed with HFRS (confirmed with positive IgG and IgM antibodies against epidemic hemorrhagic fever virus) after hospitalization (December 21, 2021). During the examination, his urinary protein was 3 + , his serum urea was 15.9 mmol/L↑ (reference range 3.1–8.0), and his creatinine was 612.2 µmol/L↑ (reference range 57.0–97.0). After hemodialysis and systemic treatment, he was discharged with improvement. However, ever since, the patient had felt sluggish all the time and had less appetite than before, without palpitation, sweating or other symptoms. However, he did not pay much attention to this because he thought it was the after-effects of HFRS. More than 20 days ago, the patient developed nausea, vomiting, poor appetite, dizziness, and fatigue without obvious inducement. After being hospitalized at a local hospital, his blood sodium concentration was 119.2 mmol/L, and he was given supplemental sodium and drugs to inhibit gastric acid secretion (reported by the patient). After treatment, his blood sodium concentration recovered to 131.5 mmol/L, and nausea and vomiting eased, but his appetite was still poor. Then, he was discharged. Approximately 1 week later, nausea and vomiting occurred again, and his blood sodium concentration fell to 126.4 mmol/L again. After sodium supplementation, his blood sodium concentration was restored to 135.3 mmol/L in the outpatient department (reported by the patient). Throughout the course of his illness, his weight gradually dropped by about 3 kg over two years, and he had no other symptoms, such as cold intolerance or loss of libido. For further treatment, he was hospitalized in our department.

On physical examination, he presented with normal vital signs (Body temperature 36.5 °C, Pulse 67 beats/min, Respiration 19 breaths/min, Blood pressure 140/90 mmHg), and his weight, height, and body mass index were 56 kg, 164 cm, and 21 kg/m^2^, respectively, with no obvious abnormalities in the eyebrows, pubic hair, or armpit hair or no other positive signs.

**Fig. 1:**
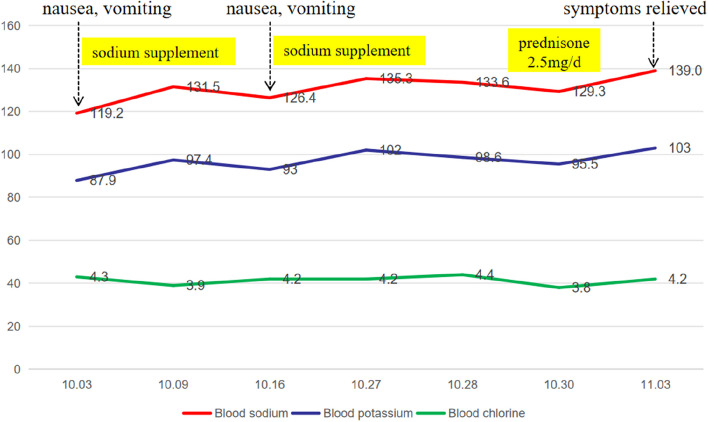
Changes in the serum electrolytes of the patient

According to the auxiliary examination, his routine blood test was normal (eosinophils 0.13 × 10^9/L [reference range 0.02–0.52]), renal function was normal (urea 3.9 mmol/L [reference range 3.1–8.0], blood creatinine 90.5 μmol/L [reference range 57.0–97.0]), and his urine specific gravity was 1.017, without any significant anomalies in other examinations, such as routine stool tests, liver function tests, blood lipids, fasting blood glucose, cardiac troponin, brain natriuretic peptide, tumor markers, or hemoglobin A1c. The 24-h urine output during hospitalization was 1280 to 2670 ml. When the patient's blood sodium concentration was 133.6 mmol/L, his urine sodium concentration was 73.4 mmol/L, and the changes in serum electrolytes are shown in Fig. 1.

He had no signs of hypothyroidism or hypogonadism. Consistently, his blood levels of thyroxine (TSH 6.050 mIU/L 0.35–5.1, FT3 3.43 pmol/L 2.76–6.45, FT4 5.91 pmol/L 11.2–23.81), testosterone (2.08 ng/ml 1.75–7.81), growth hormone (GH, 1.25 µg/L 0–2), prolactin (7.91 µg/L 2.64–13.13), luteinizing hormone (4.44 IU/L 1.24–8.62), and follicle stimulating hormone (6.16 IU/L 1.27–19.26) were all normal. However, both the plasma cortisol and the 24-h urinary-free cortisol were somewhat low, with plasma cortisol of 4.84–5.67 µg/dl at 8 AM and 24-h urinary-free cortisol of 22.8 µg (reference range 50–437 µg/24 h), and insulin tolerance test (ITT) showed no response of cortisol (peak cortisol level was 5.86 pg/ml) (As shown in Tables 1 and 2).

**Table 1:** Results of the plasma adrenocorticotropin (ACTH) and plasma cortisol (PTC) measurements

Date 2023	ACTH(pg/ml) Ref:7.2–63.4	8AM PTC (ug/dl) Ref:4.26–24.85	4PM PTC (ug/dl) Ref:2.9–17.3	0AM PTC (ug/dl)	24 h urinary-free cortisol (ug) Ref:50–437
October 10th	21.4	5.67			
October 27th	23.5	4.84	3.16	1.38	22.8

**Table 2:** Insulin tolerance test (the patient weighed 56 kg and was given an intravenous insulin injection of 5.6 U)

Time		PTC pg/ml	VBG mmol/L	ACTH ng/L	FBG mmol/L	BP mmHg	HR (BPM)
8:20	-30 min		5.20		5.2	126/88	68
9:02	0 min	5.61	4.72	24.7	4.7	127/86	63
9:07	5 min(perspire)				4.4	131/85	66
9:17	15 min(palpitations)	5.46	2.50	21.8	2.6	119/87	65
9:35	30 min	5.67	4.90		4.9	125/85	81
10:05	60 min	5.86	6.00		6.0	130/85	75

*Abbreviations: PTC* Indicates plasma cortisol, *VBS* Venous blood glucose, *ACTH* Adrenocorticotropin, *FBG* Fingertip blood glucose, *BP* Blood pressure, *HR* Heart rate, *BPM* Beats per minute

**Fig. 2:**
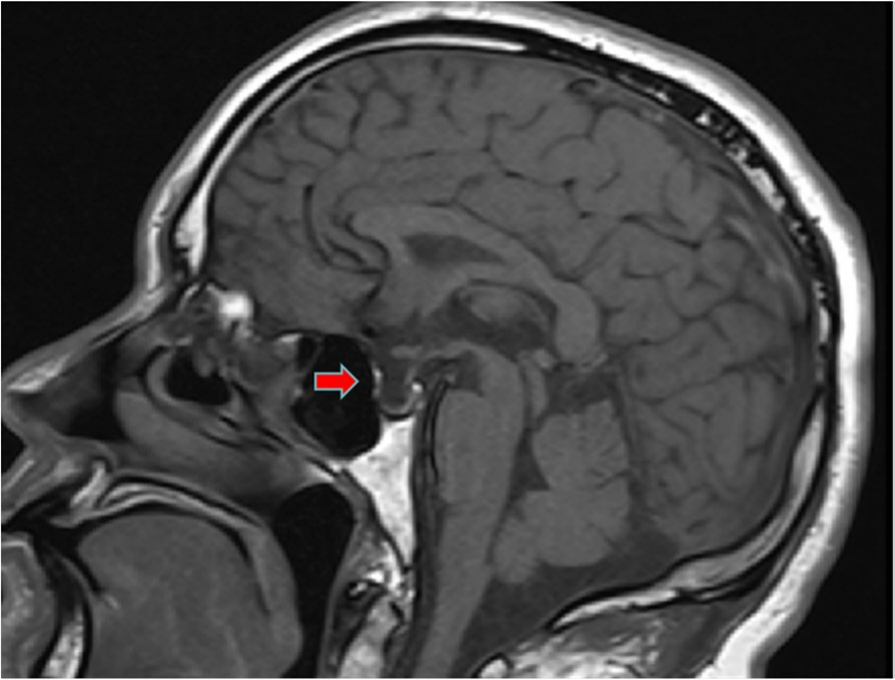
An empty sella on enhanced magnetic resonance imaging of the pituitary

Similarly, enhanced magnetic resonance imaging (MRI) of the pituitary showed an empty sella (Fig. 2). A CT scan of the adrenal gland and electrocardiogram showed no obvious abnormalities. Gastroscopy suggested erosive gastritis. Chest and abdominal CT showed scattered intrahepatic cysts or hemangiomas.

### Diagnosis and treatment process

The patient had no history of special diseases or drug use, and no significant abnormalities were found via gastroscopy, tumor marker analysis or chest or abdominal CT examinations after admission. After excluding other potent causes of nausea, such as drug sources, physical disease, potential malignant tumors, and nervous system disease, the patient was considered to have hyponatremia because his symptoms could partially recover with the recovery of hyponatremia.

The patient was diagnosed with central adrenocortical hypofunction after excluding hypertonic hyponatremia, insufficient sodium intake due to digestive tract diseases, digestive tract loss, brain salt consumption syndrome, increased extracellular fluid, antidiuretic hormone secretion disorder syndrome, hypothyroidism and other diseases that may lead to hyponatremia. His blood sodium concentration was 133.6 mmol/L, his urine sodium concentration was 73.4 mmol/L, his serum cortisol concentration was low many times, and his ACTH concentration was improperly at a normal level. Moreover, there was no response of cortisol in the ITT (for a peak cortisol level of 5.46 pg/ml). In addition, pituitary MRI indicated a vacuolar sella that also supported central adrenocortical hypofunction. This patient’s testosterone, growth hormone, prolactin, luteinizing hormone, or follicle stimulating hormone were normal. He had a mildly elevated TSH level and decreased FT4 level, but he had less appetite for the past 2 years, repeated nausea for more than 20 days, and didn’t had any symptoms associated with hypothyroidism. In addiction, his testosterone, prolactin, luteinizing hormon, and follicle stimulating hormone were all normal. Thus, insufficient T4 reserve due to insufficient iodine intake might mainly contribute to his abnormal thyroid function, which might be confirmed by further follow-up. Therefore, he was considered to have isolated ACTH deficiency at first. After admission, 2.5 mg of prednisone was administered orally at 8 AM daily. Three days later, his blood sodium concentration recovered to 139 mmol/L, his fatigue and appetite loss were completely relieved, and his spirits were good. Five months later, his thyroid function did improve (TSH 5.24 mIU/L 0.35–5.1, FT3 3.69 pmol/L 2.76–6.45, FT4 10.4 pmol/L 11.2–23.81), but TSH level was still mildly elevated and FT4 level was decreased. Meanwhile, the measurements of insulin-like growth factor binding protein-3 (IGFBP-3) and insulin-like growth factor 1 (IGF-1) were supplemented for him, and they were both decreased (IGFBP-3 3.02 µg/ml, reference range 3.5–6.7; IGF-1 49.72 ng/ml, reference range 87–234). Thus, he was finally diagnosed as partial hypopituitarism (ACTH deficiency, TSH deficiency, and GH deficiency). Since he was in excellent health, thyroid hormone and growth hormone were not supplemented for him.

## Discussion

To the best of our knowledge, this is the first case report of partial hypopituitarism with ACTH deficiency as the main manifestation that developed as a complication of HFRS. This patient’s renal function fully recovered after HFRS, but he had fatigue and a poor appetite since then. He thought that this was a “sequelae of HFRS”, with adrenal insufficiency remaining undiagnosed. In the past 20 days, his nausea and poor appetite worsened. Until admission, he was diagnosed with central adrenal hypofunction, and his symptoms were completely relieved after he was given prednisone tablet replacement therapy. Hypopituitarism after HFRS varies in severity, thus, mild patients may remain undetected for many years, as in our patient. As recommended by the TES guidelines, a healthy individual produces approximately 5-10 mg/m^2^ body surface area, which is approximately equivalent to 15-20 mg/d hydrocortisone or 3-5 mg/d prednisolone as a total replacement dose, with hydrocortisone as the first-line replacement therapy due to its low incidence of adverse events [8, 9]. However, the patient in the present study was given a single dose of 2.5 mg prednisone 8 AM daily since hydrocortisone was unavailable, which would be be further adjusted according to his clinical status and feelings since he had partial adrenal insufficiency and a single daily dose of prednisolone was frequently prescribed for central adrenal insufficiency [10, 11].

The cause of hypopituitarism after HFRS has not been fully elucidated. The following mechanisms may exist: ① Hantavirus infections may lead to thrombocytopenia via interaction with β-3 integrins on platelets [12, 13], endothelial cell dysfunction, and other coagulation disorders, which can result in pituitary hemorrhage [14]. A study of patients who died because of HFRS showed that almost 50–100% of patients had hemorrhage and necrosis in the anterior pituitary [15, 16]. In addition, hypotension and vasospasms in the acute phase of HFRS could cause ischemic damage to the pituitary [14]. ② Hantaviruses directly invade the pituitary gland and damage secretory cells of the pituitary gland, resulting in hypopituitarism [12].③ Hantavirus infection initiates an autoimmune response, and leads to pituitaritis and eventually hypopituitarism [17, 18]. Growth-hormone deficiency mostly precedes deficiency of the gonadotropic axes, followed by dysfunction of TSH and ACTH, since these conditions are more life-threatening than other conditions [19]. Thus, few cases of isolated pituitary hormone deficiency after HFRS have been reported to be GH or gonadal hormone deficiency[20]. The patient in present study was diagnosed with partial hypopituitarism with ACTH deficiency as the main manifestation. According to reports, 80–90% of the blood supply to the pituitary is from the hypophyseal venous portal circulation, and 10–20% is from the superior and inferior hypophyseal arteries. ACTH cells are mainly distributed in the posterior median region of the distal part of the pituitary gland, where blood is supplied mainly by the short and thin portal veins. However, unlike ACTH cells, the blood of regions where other hormone-stimulating cells are distributed is likely supplied mainly by the long and thick portal veins, which may explain the ACTH deficiency as the main manifestation after pituitary ischemic injury in patients with HFRS [21, 22].

At present, among the reports of hypopituitarism caused by HFRS, most involve the deficiency of multiple anterior pituitary hormones [20, 23, 24], while panhypopituitarism is rare [12, 25]. No case of isolated ACTH deficiency has been reported. In the 2008 Marko et al. study, among the 60 surviving HFRS patients, one patient was considered to have isolated ACTH deficiency, but the details were not available. They reported that 18% (11/60) of HFRS survivors had hormone deranging 3.7 ± 0.5 years(median 2 years, 6 months to 11 years) later, and 10% (6/60) of them had cortisone deficiency, one of whom had isolated ACTH deficiency and none had antidiuretic hormone deficiency [20]. Another study showed that 72–76% of patients with HFRS had anterior pituitary lobe necrosis [15]. As reported almost 70% of patients with hypopituitarism after HFRS exhibit an empty sella or low sellar density on CT or MRI of the pituitary [23]. Lee et al. reported that among those who died in the hypotensive phase of HFRS, 60% had hemorrhage and necrosis of the adenohypophysis, while almost all of those who died in the oliguric phase had hemorrhage and necrosis of the adenohypophysis [26]. The duration of hypopituitarism after HFRS was long, and the onset of this disease could be in the acute stage of hemorrhagic fever, or more than 10 years later onset, which may be related to the varying degrees of damage to the pituitary gland caused by hantavirus, the characteristics of the pituitary gland itself and the degree of patient tolerance. Pituitary function can remain normal if 50% of the gland is intact, and only when 75–80% of the pituitary is damaged will the patients develop clinical abnormalities [23]. The patient in the present study was diagnosed with partial hypopituitarism at present, but close follow-up is needed since his pituitary function may further worsen or gradually develop panhypopituitarism in the long term.

The present case is rare, and the patient received effective treatment. However, some limitations in this case report must be addressed. Firstly, since the patient, a 55-year-old man, suffered from HRFS, he began to feel sluggish all the time and had less appetite than before. He had never used glucocorticoids or other special drugs, and had no history of trauma or surgery. Therefore, ACTH deficiency caused by glucocorticoids, other special drugs, gene mutations and so on were not considered. From his history, the ACTH deficiency was most likely to be caused by HFRS. However, unfortunately, the patient failed to detect antibodies for pituitary or ACTH, and HFRS combined with ACTH deficiency caused by immune destruction can’t be ruled out, although it may be highly unlikely. Secondly, since the patient had a normal urine output and his urine specific gravity was 1.017, his antidiuretic hormone level might be normal. But if the antidiuretic hormone level could be detected, the diagnosis would be more robust. Thirdly, the 90-min value of cortisol and the peak level of growth hormone in ITT in the present case was not measured.

In conclusion, hypopituitarism after HFRS has a long duration and varies in severity, and it is easily misdiagnosed and undetected because of its nonspecific symptoms and signs. Therefore, the possibility of hypopituitarism should be considered in every patient with a history of HFRS.

## Data Availability

No datasets were generated or analysed during the current study.
